# Ureteral Dilatation with No Apparent Cause on Intravenous Urography: Normal or Abnormal? A Pilot Study

**DOI:** 10.1155/2015/681836

**Published:** 2015-10-21

**Authors:** Vinita Rathi, Sachin Agrawal, Shuchi Bhatt, Naveen Sharma

**Affiliations:** University College of Medical Sciences and Guru Teg Bahadur Hospital, Dilshad Garden, Delhi 110095, India

## Abstract

A pilot study was done in 18 adults to assess the significance of ureteral dilatation having no apparent cause seen on Intravenous Urography (IVU). A clinicoradiological evaluation was undertaken to evaluate the cause of ureteral dilatation, including laboratory investigations and sonography of the genitourinary tract. This was followed, if required, by CT Urography (using a modified technique). In 9 out of 18 cases, the cause of ureteral dilatation on laboratory investigations was urinary tract infection (6) and tuberculosis (3). In the remaining 9 cases, CTU identified the cause as extrinsic compression by a vessel (3), extrinsic vascular compression of the ureter along with ureteritis (2), extrinsic vascular impression on the right ureter and ureteritis in the left ureter (1), ureteral stricture (2), and ureteral calculus (1). Extrinsic vascular compression and strictures did not appear to be clinically significant in our study. Hence, ureteral dilatation without any apparent cause on intravenous urogram was found to be clinically significant in 12 out of 18 (66.6%) cases. We conclude that ureteral dilatation with no apparent cause on IVU may indicate urinary tract tuberculosis, urinary tract infection (*E. coli*), or a missed calculus. Thus, cases with a dilated ureter on IVU, having no obvious cause, should undergo a detailed clinicoradiological evaluation and CTU should be used judiciously.

## 1. Introduction

Ureteral dilatation, showing no apparent cause, is frequently observed in Intravenous Urography (IVU) done using ionic contrast, with or without the use of an abdominal binder, especially involving the right ureter. Spiro and Fry had reported that minor dilatation of the ureter is considered to be the residue of past pregnancy changes and of no significance [1].

Experimental studies have demonstrated that inflammatory changes in the wall of the ureter, as well as bacterial toxins, are associated with marked loss of ureteral muscle tone which is at least partly responsible for the dilatation [1]. According to Flower, the radiologist's inability to demonstrate reflux up a dilated ureter should not mislead him into suggesting that it is obstructed, as this unfortunate misinterpretation may lead to a needless or incorrect operation [2]. The concept of the “nonobstructed, nonrefluxing wide ureter” implies that the primary defect, either congenital or acquired, lies in the wall of the ureter [2].

Assessing the cause of ureteral dilatation may play an important role in influencing patient management. Ultrasonography (US) and MR imaging have been used to compensate for the limitations of IVU in the evaluation of ureters, as they do not involve ionizing radiation. However, while large portions of the ureters may not be visualized at US due to bowel gases, obesity, and so forth, MR imaging may not demonstrate ureteral calculi.

CT Urography (CTU) can clearly delineate obscure causes of ureteral dilatation, for example, presence of minute stones, radiolucent stones, or even a recently passed out stone that can be confirmed on CTU [3]. It can be used to depict unsuspected extraurinary disease [4]. Inflammatory processes adjacent to the ureter, such as appendicitis or diverticulitis, which may impair ureteral peristalsis and result in ureteral dilatation, can be identified on CTU [3]. CTU can even detect subtle benign abnormalities (e.g., ureteritis cystica) and urothelial carcinoma causing ureteral dilatation [5].

The aim of this pilot study was to assess the significance, if any, of ureteral dilatation (without any obvious cause) seen on IVU in a developing country. A clinicoradiological evaluation was done, including modified CT Urography in selected cases.

## 2. Materials and Methods

### 2.1. Patient Selection

Between November 2011 and January 2013, 18 adults showing no obvious cause of ureteral dilatation on IVU underwent a clinicoradiological evaluation. This pilot study was approved by the Institutional Ethics Committee and a written informed consent was taken from all cases. 14 cases had presented for IVU with renal calculus disease and 4 with unilateral flank pain of unknown cause.

IVU had been conducted using 40 mL ionic contrast meglumine/sodium diatrizoate (Urografin 76%, Schering AG, Germany) and abdominal binder was not applied in any of the cases.

Intravenous urograms with any obvious cause of ureteral dilatation, for example, calculus, stricture, ureterocele, megaureter, pelvic tumors, lower urinary tract obstruction, and vesicoureteral reflux, were excluded. Cases with a known cause of ureteral dilatation, for example, history of recent passage of calculus; pregnancy in the last three months, surgery involving the ureter, history of diabetes mellitus, diabetes insipidus, estrogen therapy, analgesic abuse (which causes papillary necrosis), and intake of diuretic, were also excluded.

### 2.2. IVU Evaluation

On IVU, the ureter was considered dilated if the transverse ureteral diameter was >7.0 mm at the site of maximum dilatation [1, 6], involving at least a 10 cm long segment of the ureter on supine IVU films, and the dilatation persisted in two or more radiographic exposures taken at an interval of at least 5 minutes [1]. The ureter was measured at three points, at least 1.0 cm apart (using electronic calipers on the workstation), at the widest part of the ureter; and a mean of the three measurements was recorded as the ureteral diameter in mm by Sachin Agrawal and Vinita Rathi (VR).

### 2.3. Investigations

All the patients underwent a number of investigations to exclude known causes of ureteral dilatation, for example, stone formation due to hypercalcemia and hyperuricemia, urinary tract infection, tuberculosis, diabetes insipidus, diabetes mellitus, and papillary necrosis, due to sickle cell anaemia. The investigations included assessment of urinary pH, urine specific gravity, routine and microscopic examination of urine, urine culture, microscopic examination of urine for acid-fast bacilli (AFB), urine culture for* M. tuberculosis*, haemogram with ESR, peripheral smear examination to exclude sickle cell anaemia, serum uric acid, serum calcium, fasting blood sugar, Mantoux test, and chest radiograph and sonography of the genitourinary tract (to exclude ureteral calculi and tumors involving pelvic organs).

### 2.4. Study Design

Those cases, in which the cause of ureteral dilatation on IVU was apparent on laboratory investigations and sonography, were excluded from further examination to avoid undue exposure to radiation. The remaining cases underwent CT Urography, within one week of IVU, to identify any other obscure cause of ureteral dilatation.

Voiding cystourethrography or cystourethroscopy was done, if clinically indicated. All cases were followed up clinically by the surgeon (Naveen Sharma) and on sonography till the end of the study period (i.e., 3 months to 1 year).

### 2.5. CT Scan Protocol

CT Urography was carried out by a modified technique on a 64-slice scanner (Somatom Definition AS, Siemens, Forchheim, Germany). Axial sections of 3.0 mm slice thickness were acquired and reconstructed at 1.0 mm interval. Scans were started one vertebral level above the region of maximum dilatation of the ureter on IVU and continued till the level of the ischial spines.

A noncontrast CT scan was performed at 120 kVp and 165 mAs to identify any small radiopaque calculus in the ureter. If the cause of ureteral dilatation was identified on noncontrast scans, the subsequent phases of CT Urography with contrast were abandoned, to avoid unnecessary irradiation.

100 mL nonionic contrast iohexol 300 mg I/mL (Omnipaque 300 GE Healthcare) was injected intravenously, and nephrogenic phase scans were acquired after a delay of 80–100 seconds at 120 kVp and 165 mAs, to evaluate the ureteral walls and periureteral disease.

Excretory phase of CT Urography was performed at low dose, that is, 120 kVp and 100 mAs, to reduce the radiation exposure.

The cause of ureteral dilatation was evaluated on CT Urography by two radiologists, VR and Shuchi Bhatt, independently. Ureteritis was diagnosed in the presence of diffuse, mild to moderate, circumferential ureteral wall thickening and enhancement associated with periureteric stranding [7]. Vascular compression was defined as extrinsic pressure effect on the ureter at the level where it crosses the iliac vessels [8], resulting in an abrupt change in the caliber of the ureter.

## 3. Results

The study comprised 18 cases (12 females and 6 males) with age ranging between 18 and 60 years and the mean age being 32.2 years. 50% of the cases were less than 30 years of age.

Out of the 12 females, 10 were in the reproductive age group, while 2 were postmenopausal. In the former group, 8 were multiparous (having 2 to 7 issues) and 2 were nulliparous.

On IVU, 35 out of 37 ureters were measured (1 case had a duplicated left ureter, and in two cases, one of the ureters was not opacified due to complete obstruction by a renal calculus). The maximum ureteral diameter on IVU ranged from 3.0 to 20.0 mm. 22 out of 35 ureters were dilated: 15 on the right and 7 on the left. Ureteral diameter was normal (<7.0 mm) in 13 of the 35 ureters: 2 on the right and 11 on the left. In both sexes, ureteral dilatation was more frequent on the right side: in 10 out of 15 (66.66%) females and 5 out of 7 (71.43%) males. 12 ureters were dilated on the symptomatic side and 8 ureters were found dilated on the urinary pH and specific gravity and routine examination for albumin and sugar were normal in all cases. Details of the urine analysis done for assessing infection are shown in Table 1. Total leucocyte count, differential leucocyte count, serum uric acid, and fasting blood sugar levels were also normal in all cases. Seven cases had anemia, of which 4 had iron-deficiency anaemia. ESR was elevated in 6 cases. Four cases had a positive Mantoux test and three cases had hypocalcemia. Chest radiographs were normal in all cases except Case 14, in which left-sided pleural thickening was seen.

Sonography of the genitourinary tract revealed renal calculus disease causing hydronephrosis in 13 out of 18 cases. Hydroureter was found in 5 of the 18 cases, which had shown a dilated ureter on IVU. The cause of hydroureter could not be identified on sonography. Vesicoureteral reflux was absent on colour Doppler in all cases. Urinary tract infection/tuberculosis was diagnosed in 8 out of the mentioned 13 cases on laboratory investigations, while the remaining 7 cases underwent CT Urography.

Based on the laboratory investigations and sonography, the cause of ureteral dilatation was identified in 9 out of 18 (50%) cases. Ureteral dilatation was attributed to urinary tract infection in 6 cases (pus cells on urine microscopy: 3 cases; positive urine culture: 3 cases) and urinary tract tuberculosis in 3 cases. Thus, excluding these (6 + 3 = 9 cases), the remaining 9 cases underwent a modified CT Urography to evaluate the cause of ureteral dilatation. The causes of ureteral dilatation revealed on CT Urography are listed in Table 2. In Case 1, noncontrast CT detected a ureteral calculus as a cause of ureteral dilatation; hence, contrast phase and excretory phase of CTU were not performed.

Voiding cystourethrography was done in Case 12 to evaluate the cause of the left-sided ureteritis and chronic pyelonephritis of the left kidney; but no vesicoureteral reflux was demonstrated. Ureteroscopy was not done to evaluate ureteral stricture in Cases 7 and 8, as it was not indicated clinically.

Ureteral dilatation caused by extrinsic compression by iliac arteries (seen on CTU) in four ureters (Cases 5, 6, 12, and 18) and the strictures reported in Cases 7 and 8 (on CTU) showed no clinical significance, since after undergoing pyelolithotomy these patients were asymptomatic during the follow-up period. Thus, excluding these six cases, a dilated ureter on IVU was found to be clinically significant in 12 out of 18, that is, 66.6% cases.

## 4. Discussion

The causes of ureteral dilatation are divided into obstructive and nonobstructive. Dilatation of the right ureter with associated urinary tract infection in previously gravid women is often ascribed to pressure upon this structure by an incompletely involuted, aberrant, dilated, right ovarian vein [8].

With the advent of multislice CT (MSCT) scan in developing countries and its inherent advantages in detecting urinary and extraurinary causes of ureteral dilatation, we performed a pilot study to assess the reason for ureteral dilatation on Intravenous Urography in those cases where this was not apparent. However, considering the lack of easy availability and high cost of MSCT in developing nations, we used a detailed clinicoradiological evaluation including laboratory investigations and CT Urography (using a modified technique), if necessary.

The present study had been conducted in patients with renal calculi (14) or undiagnosed flank pain (4), who presented for IVU to the department of radiodiagnosis. Only 18 out of 200 IVU cases presenting during the study period had dilated ureters with no apparent cause.

The right ureter was dilated in 90% (9 out of 10) parous women in our study. Our findings were similar to Flower who had stated that dilated ureters on the right, particularly, are sometimes seen in adult parous females. A combination of hormonal effect and past or present urinary tract infection had been postulated and evidence of obstruction and reflux could not be demonstrated [2]. On IVU of 26 parous women, Dure-Smith also found the width of the right ureter was greater than the left one in 76% of cases [9]. In our study in male patients too, dilatation was more commonly seen in the right ureter.

Ureteral dilatation with no obvious cause on Intravenous Urography was found to be clinically significant in 12 out of 18 (66.6%) cases. In 8 out of these 12 cases, it was associated with urinary tract infection (pus cells on urine microscopic examination: two;* E. coli* infection on urine culture: three; ureteritis on CTU: three). Identification of urinary tract infection (UTI) is important in patients with renal calculi to prevent postoperative complications and also in patients with flank pain in the absence of calculus. Thus in patients with ureteral dilatation with no obvious cause on IVU, if urinary tract infection is detected, it should be adequately treated. Zelenko et al. had stated that* E. coli*,* Pseudomonas,* and* Citrobacter* infections can impair ureteral peristalsis and cause ureteral dilatation [3]. Findings of Spiro and Fry had also suggested that ureteral dilatation is associated with infection, that is, pelvi-ureteritis, past or present [1].

**Figure 1:**
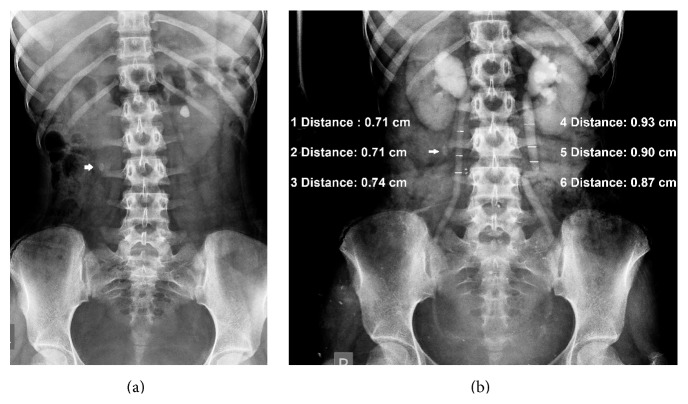
(a) Scout film shows a left renal calculus. An oval calcified node (arrow) lies outside the line of the ureter in (b). (b) Intravenous urogram shows dilated ureters with no apparent cause. Acid-fast bacilli on microscopic examination and culture of urine confirmed tuberculosis.

**Figure 2:**
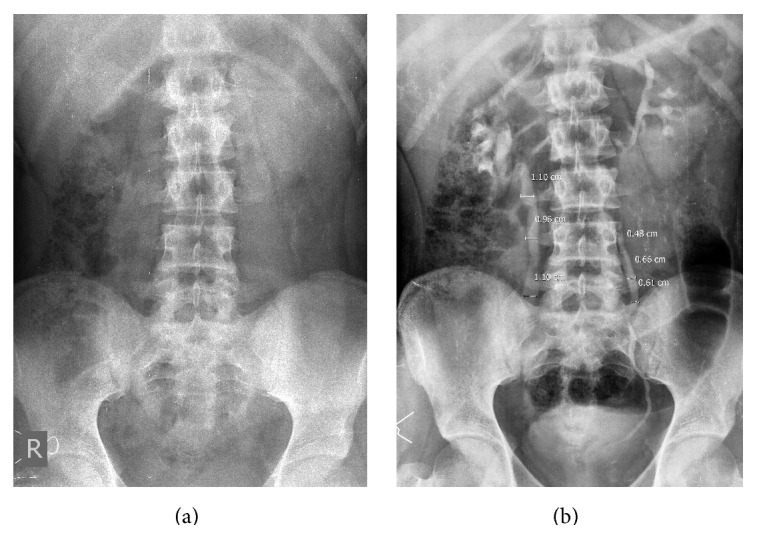
(a) Scout film: male patient with right-sided flank pain. No radiopaque calculus seen. (b) IVU: right ureter was dilated, but the cause was not obvious. No vesicoureteral reflux seen on color Doppler. Urine examination revealed tuberculosis.

In 3 out of the 12 cases, dilated ureters on IVU were likely to be a manifestation of tuberculosis (confirmed on urine examination) in our study, as no other signs suggestive of tuberculosis had been seen on either IVU or sonography. In 2 out of these 3 cases (having dilated ureters with no apparent cause on IVU), patients had presented with renal calculi and IVU had been requested preoperatively (Figure 1). Hence, urinary tract tuberculosis would have remained undiagnosed and untreated in these 3 cases, if they had not undergone a detailed clinicoradiological evaluation for the cause of ureteral dilatation (Figure 2).

In one case, ureteral dilatation on IVU was due to a ureteral calculus (identified on CTU), which had been missed on a plain radiograph and not detected by sonography. CTU played a very important role in the management of this case, as ureteroscopic removal of stone was done.

**Figure 3:**
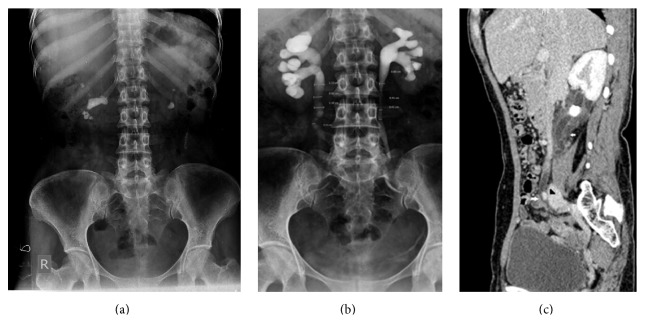
(a) Scout film: bilateral renal calculi. (b) IVU: a dilated right ureter, but the cause was not apparent. No ureteral calculus on noncontrast CT. (c) Contrast-enhanced CT scan: marked wall thickening in the region of ureteral dilatation (arrow) indicating ureteritis and also an extrinsic vascular compression of the right ureter (between arrowhead and lower white arrow).

CT Urography was performed in 9 out of the 18 (50%) cases and identified the cause of ureteral dilatation in all the 10 dilated ureters (100%) (Table 2). But the cause of dilatation was clinically significant in only 4 of the dilated ureters (calculus: 1, ureteritis: 1, and ureteritis along with extrinsic compression by a vessel: 2) (Figure 3).

In another 4 ureters, CTU showed no significant cause of ureteral dilatation other than extrinsic vascular compression. According to Chait et al., minor degrees of obstruction of the ureter caused by iliac vessels do not appear to be clinically significant [8].

In two cases, stricture was the possible cause of ureteral dilatation on CTU, but these patients developed no fresh clinical complaints till one year, after undergoing surgery for renal calculus. Thus, these strictures were not considered clinically significant. Ureteroscopy was not warranted clinically and hence was not performed. However, a prolonged follow-up is required to assess the significance of the ureteral strictures seen on CTU in these two cases.

## 5. Conclusions

In developing countries, availability and cost of MSCT are major limitations. Hence cases having a dilated ureter with no apparent cause on IVU should undergo a detailed clinicoradiological evaluation that includes laboratory investigations to identify the cause. CT Urography should be used judiciously and the technique should be modified (e.g., restricting scan length to identify cause of ureteral dilatation and using low dose technique) to limit the radiation dose.

## Figures and Tables

**Table 1:** Urine analysis (*n* = 18).

Investigation	Abnormal	Normal
Microscopic urine examination		
Pus cells^*∗*^	8	10
RBCs	3	15
Urine culture	3 (*E. coli* > 100000 CFU/mL)	15
Microscopic examination for acid-fast bacilli (AFB)	3	15
Culture for *Mycobacterium tuberculosis*	3	15

^*∗*^1 case showed microscopic field full of pus cells; 7 cases showed 4–10 pus cells/HPF.

Urinary tract infection was found in 8 patients with a dilated ureter, out of which 3 showed growth of *E. coli* on urine culture, while in 2 cases *Mycobacterium tuberculosis* was isolated on culture (1 patient with genitourinary tuberculosis had no pus cells in urine).

**Table 2:** Causes of ureteral dilatation on CT Urography (*n* = 9).

Number	Right ureter	Left ureter
Case 1	Ureteral calculus	Normal ureter
Case 2	Extrinsic vascular impression (internal iliac artery); ureteritis	Normal ureter
Case 4	Not assessed^*∗*^	Extrinsic vascular impression (common iliac artery); ureteritis
Case 5	Extrinsic vascular impression (common iliac artery)	Normal ureter
Case 6	Extrinsic vascular impression (bifurcation of common iliac artery)	Normal ureter
Case 7	Ureteral stricture at external iliac artery origin level	Normal ureter
Case 8	Ureteral stricture at lumbosacral junction level	Normal ureter
Case 12	Extrinsic vascular impression (common iliac artery)	Ureteritis
Case 18	Extrinsic vascular impression (common iliac artery)	Normal ureter

^*∗*^Case 4: right ureter was not assessed on CT Urography as it was not visible on Intravenous Urography and hence not dilated.
